# Paracentral acute middle maculopathy following COVID-19

**DOI:** 10.5935/0004-2749.2023-0064

**Published:** 2023

**Authors:** Aluisio Rosa Gameiro Filho, Rafael Godoy, Angela Rees, Simona Degli Esposti

**Affiliations:** 1 Retina Department, Moorfields Eye Hospital NHS Foundation Trust, London, UK; 2 Médicos de Olhos SA, Campo Largo, PR, Brazil

Dear Editor,

Coronavirus disease 2019 (COVID-19) is caused by severe acute respiratory coronavirus 2 (SARS-CoV-2). Since the outbreak of this disease, several reports of associated ophthalmological manifestations have been published. They usually occur up to two weeks after the beginning of systemic symptoms; however, in 2.26% of cases, the eye can be the site of initial COVID-19 manifestation^(1)^. Even though disorders of the ocular surface, such as conjunctivitis, keratitis, and episcleritis are the commonest findings in these patients, there are some reports of retinal involvement, mostly vaso-occlusive events. We report two cases of paracentral acute middle maculopathy (PAMM) associated with COVID-19.

## Case 1

**Figure 1:**
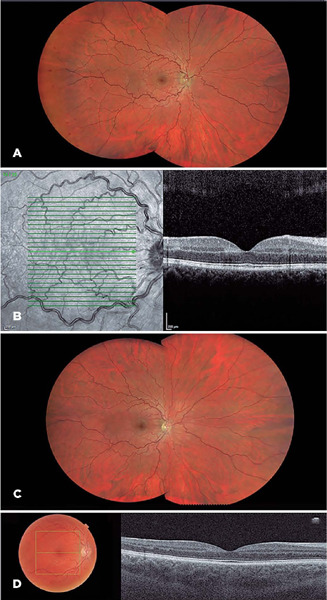
A: Fundoscopy showed increased vascular tortuosity and hemorrhages in the temporal region. B: OCT showing a hyper-reflective band in the INL suggestive of PAMM. C: Fundoscopy, 1 month after the beginning of the symptoms. D: OCT 1 month after the beginning of the symptoms. INL –inner nuclear layer, OCT–optical coherence tomography, PAMM–Paracentral acute middle maculopathy.

A 36-year-old man attended accident and emergency (A&E) complaining of painless visual reduction in his right eye associated with mild headaches, which had started 1 week before. Five days before, he had been diagnosed with COVID-19. He had received 3 doses of COVID-19 vaccine, with the last dose 2 months before the onset of symptoms. His past medical and family histories were unremarkable. The best corrected visual acuity was 20/80 in the right eye (OD) and 20/20 in the left eye (OS). His intraocular pressure (IOP) and biomicroscopy were normal. Fundoscopy showed increased tortuosity and dilatation of all branches of the retina central vein with some hemorrhages in the temporal areas, suggesting branch retinal vein occlusion (BRVO) in the OD. Fluorescein angiography revealed a delay in arteriovenous time, and optical coherence tomography (OCT) showed a hyper-reflective band in the inner nuclear layer (INL), suggestive of PAMM. After 1 month of observation, his visual acuity returned to 20/20 in both eyes. Blood test results showed normal levels of homocysteine and were negative for thrombophilia. The OCT findings also returned to normal (Figure 1).

## Case 2

**Figure 2:**
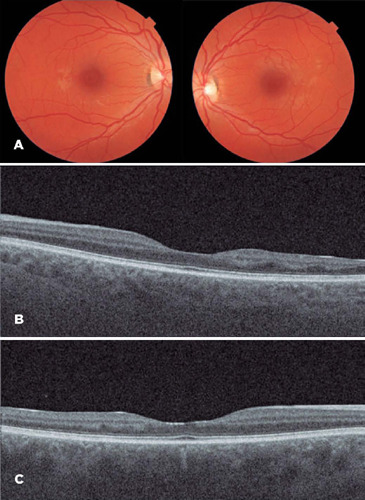
A: Normal fundoscopy 2 years after the acute event. B and C: OCT showing temporal thinning of the right (B) and left (C) inner nuclear layers, compatible with late PAMM.

A 30-year-old female patient attended A&E complaining of floaters and scotoma in both eyes. She had recently been diagnosed with COVID-19 and was still breathless and weak on minimal exertion. Her visual acuity was 20/15 and 20/20 in OD and OS, respectively. Biomicroscopy and fundus examination were normal, and the patient was referred for a neuro-ophthalmological examination. OCT was performed, revealing PAMM in both eyes. Two years later, the patient has bilateral retinal INL thinning compatible with late PAMM (Figure 2).

COVID-19 is associated with a state of hyper-inflammation and hypercoagulability, causing thrombotic complications in several organs and tissues. First described by Sarraf in 2013, PAMM is a spectral-domain OCT (SD-OCT) finding characterized by band-like lesions visible at the level of the INL. It is considered the acute phase of ischemia in the intermediate and deep capillary process^(2)^. It usually affects patients about 50-60 years old, with no gender predilection. This condition was previously associated with COVID-19 infection^(3)^ and patients who received COVID-19 vaccination^(4)^. COVID-19 is thought to cause vascular degeneration in the eye through several mechanisms: by direct viral entry into retinal cells via angiotensin-converting enzyme (ACE-2) receptors causing endothelial cell damage and thromboinflammation. When the virus-ACE2 receptor connection is established, the outer part of the receptor is released, leading to a downregulation of the receptor, causing a dysregulation in the renin-angiotensin system, affecting vascular constriction, proliferation, and the inflammatory status of the vascular tree^(5)^; also, by downregulation of endothelial nitric oxide synthase, and abnormal vascular endothelial growth factor expression, inducing a cytokine storm^(5)^; by inducing a deregulation of the autonomic system which controls choroidal blood flow^(3)^; and through coagulation disorders, which can be detected by increased D-dimer levels^(4)^.

The ophthalmologist should be aware of the association of COVID and COVID-19 vaccination, with retinal vaso-occlusive conditions, particularly in young patients with no risk factors for thromboembolic and vascular events.
